# Allied health research positions: a qualitative evaluation of their impact

**DOI:** 10.1186/s12961-016-0166-4

**Published:** 2017-02-06

**Authors:** Rachel J. Wenke, Elizabeth C. Ward, Ingrid Hickman, Julie Hulcombe, Rachel Phillips, Sharon Mickan

**Affiliations:** 1 Clinical Governance, Education and Research (Allied Health), Gold Coast Health, 1 Hospital Boulevard, Southport, Queensland 4215 Australia; 2 School of Allied Health Sciences, Griffith University, Parklands drive, Southport, Queensland 4215 Australia; 3 Department of Health, Centre for Functioning and Health Research, Metro South Hospital and Health Service, Eight Mile Plains, Brisbane, Queensland Australia; 4 School of Health & Rehabilitation Sciences, The University of Queensland, Level 7, Therapies Building, Services Rd, St Lucia, 4072 Queensland Australia; 5 Department Nutrition and Dietetics, Princess Alexandra Hospital, Ground Floor, Building 15, Ipswich Rd, Woolloongabba, Queensland 4102 Australia; 6 Allied Health Profession’s Office of Queensland, Department of Health, Level 1, 15 Butterfield Street, Herston, Queensland 4006 Australia; 7 Ipswich Hospital, West Moreton Hospital and Health Service, PO Box 73, Ipswich, Queensland 4305 Australia

**Keywords:** Allied health, Research personnel, Capacity building

## Abstract

**Background:**

Research positions embedded within healthcare settings have been identified as an enabler to allied health professional (AHP) research capacity; however, there is currently limited research formally evaluating their impact. In 2008, a Health Practitioner industrial agreement funded a research capacity building initiative within Queensland Health, Australia, which included 15 new allied health research positions. The present project used a qualitative and realist approach to explore the impact of these research positions, as well as the mechanisms which facilitated or hindered their success within their respective organisations.

**Methods:**

Forty-four AHP employees from six governmental health services in Queensland, Australia, participated in the study. Individual interviews were undertaken, with individuals in research positions (n = 8) and their reporting line managers (n = 8). Four stakeholder focus groups were also conducted with clinicians, team leaders and professional heads who had engaged with the research positions.

**Results:**

Nine key outcomes of the research positions were identified across individual, team/service and organisational/community levels. These outcomes included clinician skill development, increased research activity, clinical and service changes, increased research outputs and collaborations, enhanced research and workplace culture, improved profile of allied health, development of research infrastructure, and professional development of individuals in the research positions. Different mechanisms that influenced these outcomes were identified. These mechanisms were grouped by those related to the (1) research position itself, (2) organisational factors and (3) implementation factors.

**Conclusions:**

The present findings highlight the potential value of the research positions for individuals, teams and clinical services across different governmental healthcare services, and demonstrate the impact of the roles on building the internal and external profile of allied health. Results build upon the emerging evidence base for allied health research positions and have important implications for a number of stakeholders (i.e. individuals in the research positions, AHPs and their managers, university partners and state-wide executives). Key recommendations are provided for all stakeholders to enhance the ongoing impact of these roles and the potential advocacy for additional positions and resources to support them.

**Electronic supplementary material:**

The online version of this article (doi:10.1186/s12961-016-0166-4) contains supplementary material, which is available to authorized users.

## Background

Allied health is the third largest workforce in healthcare and is comprised of a diverse number of professions, including physiotherapy, exercise physiology, social work, psychology, speech pathology, dietetics, occupational therapy, radiography and podiatry, among others. Building the capacity of allied health professionals (AHPs) in healthcare to undertake research is considered to be an international priority [1–4]. Benefits of health professionals engaging in research span beyond the individual’s professional development. Health organisations with a strong research culture have been associated with greater service efficiencies and reduced patient mortality and morbidity [5]. Barriers to AHPs undertaking research are widespread and include reduced clinician time and lack of skills and confidence [6–9]. Research capacity building is defined as “*a process of individuals and institutional development which leads to higher levels of skills and greater ability to perform useful research*” ([10], p. 1321). Research capacity building strategies evaluated within AHPs include targeted research skills training, funding bursaries and mentoring [6–9]. Embedding dedicated research positions within healthcare organisations is also cited as a strategy to promote AHP research capacity [1, 11–13].

Dedicated research positions in healthcare settings to support AHPs to undertake research are becoming more common, particularly within metropolitan tertiary health services, with the majority of prevalence data emerging from Australia. For example, a survey of 520 AHPs across all hospitals in Victoria, Australia, revealed that approximately a third of respondents had access to a co-located research position [14]. Of these respondents with access to an allied health research position, 96% were based in a metropolitan area [14]. The reduced accessibility of research positions in rural areas was further highlighted in a recent Australian study which described that the lack of accessible research positions may hinder the research activity of AHPs working in rural Queensland [15]. Further investigation into the impact of research positions on allied health research capacity across different organisations is needed to confirm evidence for their benefit and support future investment.

A recent systematic review revealed that there are few empirical studies formally evaluating the impact of allied health research positions within healthcare [16]. The current evidence base indicates that research positions embedded within healthcare organisations can impact individual and team research skills and research participation of AHPs [14, 17, 18]. Victorian AHPs found that respondents with access to a dedicated research position reported significantly greater research activity and involvement in writing/dissemination, funding and data collection compared to those without access [14]. A mixed methods study also explored the impact of a research position appointed to provide research support for nurses, midwives and AHPs within a United Kingdom healthcare trust [17]. The study reported positive changes to research culture, increased publications, presentations and changes in practice as a result of the position. The authors also highlighted that leadership support within the organisation positively influenced research engagement [17]. While these studies provide emerging evidence of the potential value of research positions embedded within healthcare on increasing AHP research skills and participation, it remains unclear what the broader impact of these positions is across different organisational contexts. Further, the mechanisms which facilitate or hinder their success are also largely unknown.

### Local context

A unique opportunity to potentially address some of these gaps in the literature was created by an Australian state Government Health sector following the establishment of 15 Health Practitioner research positions in 2008 for AHPs. These continuing research positions were funded to build research capacity of allied health clinicians, alongside a continuing Health Pracitioner Research Grant Scheme for AHPs. To date, the outcomes of the research positions have been monitored through annual reporting of key performance indicators, including grant funding, peer reviewed presentations/publications, number of higher degree research students being supervised, education and training, and participation in collaborative networks [1]. These key performance indicators do not capture the full impact of the research positions, nor the underlying mechanisms that facilitated or hindered the success of the roles. Further understanding is needed regarding the impact and outcomes of these research positions within their respective organisations, and the underlying mechanisms that facilitated or hindered their outcomes.

## Methods

### Aims

The present project had two primary objectives, namely (1) to identify and explore the impact of government-funded research positions on building allied health research capacity within their organisational context, and (2) to describe mechanisms that enable and/or hinder the impact of the research positions in building allied health research capacity.

### Design

The study employed a qualitative methodology, informed by a realist approach [19]. Interviews commenced with individuals in the research positions and their managers, together with key stakeholders identifying key impacts of the research position roles. Interviewees were then facilitated to discuss how these outcomes had been achieved and what factors enabled and hindered the outcomes identified. The researchers wanted to understand which mechanisms supported key outcomes and in what circumstances they were most enabling.

### Participants and recruitment

In July 2015, the Directors of Allied Health of nine state healthcare organisations that currently employed a research position were invited to participate by email. Six health services indicated interest in participating in the project. Each of these health services included one tertiary or a large regional hospital with several associated subacute and/or community-based facilities integrated within a single management structure in the same geographical region. Ethical clearance and site-specific assessment approval (HREC/15/QGC/210) was sought and granted from all six sites to recruit participants from three groups, namely (1) research positions, (2) reporting line managers, and (3) stakeholder participants.

#### Research positions

At the time of recruitment, 10 individuals were employed in the research positions within the six participating sites. Purposive sampling was used to ensure an even representation of research positions across all six sites. Eight were invited to participate and all provided their consent. All participants in the research positions were health service employees and three also carried conjoint positions with local universities. Mean duration in the role was 4.1 years (SD = 1.6, range = 1 to 5 years), with mean years since receiving a PhD being 11.3 years (SD = 6.3, range = 3 to 22 years, n = 7). Four of the eight research positions were full time, the remaining five were part time positions (range 2.5–4 days per week). Background professions of the research positions included occupational therapy (n = 1), nutrition and dietetics (n = 2), exercise physiology (n = 1), physiotherapy (n = 1), speech pathology (n = 1), psychology (n = 1), and medical science (n = 1). Two of the eight research positions were professorial appointments.

#### Reporting line managers

After each individual in the research positions consented to participate, their current or previous line managers were invited to participate. All invited managers provided consent to participate. On average, these managers had been supervising the research position for 2.2 years (SD = 1.6, range = 0.5 to 5 years).

#### Stakeholder participants

Clinical and managerial staff who had worked with the research positions were invited to focus groups at four of the participating sites. These sites were purposively chosen to reflect maximum geographic diversity. From the 69 AHPs nominated from these sites, a maximum variation purposive sample of 50 were invited to participate, with 28 staff consenting to participate. Staff declined most commonly because of conflicting time priorities (n = 20), while others declined due to changed positions (n = 2). Participants came from eight different AHP backgrounds and ranged from base-grade clinicians to professional directors, with the majority being senior clinicians (Table 1).

**Table 1:** Professional background and level of experience of focus group participants

Profession	No. of participants (n)
Audiology	1
Dietetics	5
Medical Imaging	4
Occupational Therapy	6
Oral Health	1
Physiotherapy	5
Social Work	3
Speech Pathology	3
Level of experience	
Base grade	3
Senior clinician	8
Team leaders	11
Professional director/manager	6

### Data collection methods

Data was collected through 16 semi-structured interviews and four focus groups conducted by the first author (RW). The health service that was funded to conduct this research had discontinued previous research positions before the appointment of current researchers (RW and SM). Therefore, there was both a real and perceived level of independence in collecting and analysing data. RW was employed as a project officer and interviewees were aware that she had an AHP background, had previously completed clinical research and was temporarily employed in a research role. All interviewees were also informed that their responses were confidential and would not in any way impact their employment. RW did not have any existing association with any of the interviewees, with the exception of two interviewed research positions (EW and IH), who had academic involvement with RW, in regards to data analyses and write-up assistance. All interviews were audio recorded and interview notes were taken by the interviewer. To enhance reflexivity, the interviewer additionally took reflective notes after each interview [20]. This helped to sensitise the interviewer regarding how her own experiences may potentially influence interviews, and these reflections were regularly shared with another author (SM) as the interviews progressed.

One-on-one semi-structured interviews were undertaken with the research positions and their line managers at a locally convenient time and place or through the use of video/teleconference. All focus group interviews were conducted face-to-face with staff from the same health service. Interview questions were sent to participants via email approximately 1 week prior to the interview to allow time for reflection. Interview questions explored successes and achievements and mechanisms which facilitated/hindered these, as well as the impact of the roles on research capacity. The semi-structured interview guides can be found in Additional file 1.

### Data analysis

Interviews were professionally transcribed and transcripts were sent to participants to check the integrity of the data. Qualitative research analysis software NVivo [21] was used to perform a conceptual analysis of the data. Interviews were coded into common topics and categorised within themes that represented outcomes, enabling mechanisms and contexts. Two raters (RW and SM) reviewed the coding and categorisation using an iterative consensus decision-making process. While a realist evaluation typically explores the relationship between context, mechanisms and outcomes [19], similar contexts were found for each of the outcomes. As such, the results will focus on the different mechanisms that supported each outcome. Interviewees commonly described both enabling and hindering mechanisms, which were often two sides of a similar issue. For this report, mechanisms will be described positively.

## Results

### Key outcomes and mechanisms

**Fig. 1:**
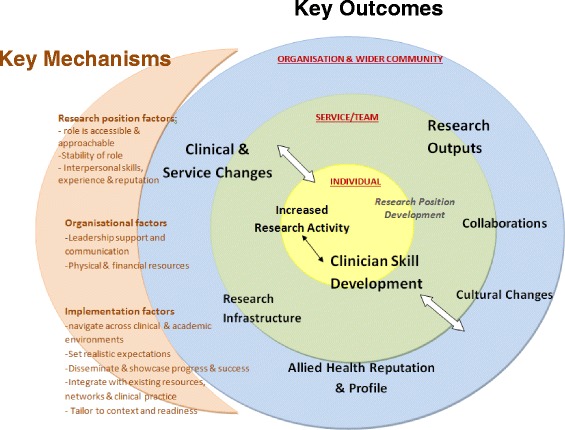
Outcomes and mechanisms of the allied health research positions

The interviews revealed nine key outcomes of the research positions as represented in Fig. 1. These outcomes influenced a number of levels within and beyond the health service, including individual, service/team and organisational/wider community levels, as represented by the three concentric circles in Fig. 1. For each of the outcomes, interviewees described specific mechanisms which either enabled or hindered the success of the outcomes. As shown in Fig. 1, these mechanisms were broadly grouped into three categories: research position factors, organisational factors and implementation factors. Table 2 describe specifically how each mechanism is linked to seven key outcomes and is presented as a matrix. As fewer mechanisms were described for the research infrastructure outcome and the professional development of the research position, these outcomes will be described in the text. Additional quotes supporting each of the outcomes and their mechanisms are found in Additional file 2. Quotes were coded with the letter “R” if they were from a research position participant, “M” for quotes from manager participants, and “F” indicating a response from a stakeholder focus group participant (i.e. clinician, team leader or professional director).

**Table 2:** Outcome and mechanisms for the allied health research positions

Factor description		Outcomes of Research Positions						
		Clinician skills development	Increased research activity	Clinical and service changes	Research outputs	Collaborations	Cultural changes	Allied health reputation and profile
Research position	Role is accessible and approachable	- Internally promote role - Be perceived as available and approachable by clinicians - Physical proximity to clinicians	Full time position		- Full time position - Balance support for clinicians and own research progress			
	Stability of role		Being in the position longer term		Position consistent over time, at right pay point	Having time in the position to build relationships		
	Interpersonal skills, experience and reputation	- Broad research experience - Willingness to move outside clinical interest and expertise - Access to research team	- Having a clinical background - Good interpersonal skills	Understanding of health service	- Being linked with a university - Track record	- Experienced with large existing networks - Trusted and respected Conjoint position		- Reputation for undertaking high quality research - Track record
Organisational factors	Leadership support and communication	Clinicians having line manager support to access training/mentoring	- Leaders allow clinicians to go offline - Research as key performance indicator or part of core business		- Management support - Promote upcoming grants - Assistance operationally managing grants		- Leaders undertake research - Research allowed to be done in work time - Existing research culture - Communicate updates to Executive	- Communicate research outputs to Executive level - Executive Director of Allied Health promotes research achievements - Research standing agenda item at meetings
	Physical and financial resources	Funding for external speakers	Physical space access to research and statistical software and expertise		- Supportive internal grant opportunities linked with RHD pathway - Administrative Officer or Research Assistant support			Organisation provides opportunities to present
Implementation factors	Integrate with existing resources, networks and clinical practice	- Access to pre-existing training programmes - Consistent time and place for learning - Embed into existing teams professional development programme - Arrange external speakers	- Clinically meaningful projects that are achievable within everyday practice - Encourage clinicians to support other projects led by more experienced researchers	Encourage projects close to practice	- Internal publishing team - Help clinicians choose PhD topics with broad clinical impact close to their practice			Research position is integrated with other established leadership meetings
	Tailor to context and readiness	- Break down research process - Tailor to needs/developmental level experience - Motivate clinicians throughout process - Encourage self-directed learning and flow on learning - Group-based learning	Respect clinician readiness, targeting those who are interested					
	Disseminate and showcase progress and success		Showcase/role model clinicians		- Support clinicians to disseminate, make it a positive experience - Showcase clinician’s PhD experiences			Provide presentations internally and externally and promoting research role
	Set realistic expectations	Awareness role includes capacity building	Set realistic expectations and timeframes with clinicians		Have strategy for type of research program and plan for dissemination		Helping clinicians see research can be attainable	Plan research in advance to maximise quality
	Navigate across clinical and academic environments			Have an understanding of health service		Respect strengths and skills of clinicians and academics		Navigate process of conference leave approval for local hospital system
	Other		Clinician persistence	Encourage clinicians to use evidence-based practice			Mentoring and upskilling team	Governance to support quality research

*PD* professional development, *KPI* key performance indicator, *RHD* research higher degree, *AO* administrative officer, *RA* research assistant

### Clinician skill development

Research positions most commonly developed clinicians’ research skills through individual mentoring and targeted team-based education. One clinician described, “*So* [the research position]*’s supported me to be able to apply for research funding, get a couple of research studies up and going, apply for ethics, get publications accepted, present research* [overseas] *and I’ve just got two new studies started up this year… I don’t think I could have done that without her*” (F4). Individual clinicians were also empowered to up skill their peers, “*…those fundamental skills in someone can instantly be passed on to someone else if they’re shown in the correct way. So that’s been a really great impact to give me the confidence to go help others start their journey…*” (F2). Having an accessible research position was acknowledged by a manager to address the commonly reported barrier of time, “*Proximity is actually really important… if they’re sitting literally outside my office… clinicians are more likely to walk in and say while I’ve got you, can we have a quick chat about x, y, z*” (M1).

### Increased research activity

The research positions were reported to increase the number of clinicians engaging in research activity, “*Getting some of those departments that weren’t doing anything to actually be doing something is a big success*” (R2). The increased activity led to a snowball effect of progressively more clinicians engaging in research, “*When I first started I think there was probably less than half a dozen people actively participating in research. I think I’ve got over 40 research projects currently in process now*” (R5). Making research projects clinically meaningful was a key enabling mechanism described as, “*It’s making it as easy for them as it possibly can be and that’s essential. It’s making it clear to them that you’re trying to answer something that's going to help them in the long run*” (R1).

### Clinical and service changes

The research positions were described to have contributed to clinical practice changes that improved patient and service outcomes. One manager explained, “*… they’ve actually changed their clinical procedures and they’re doing it very differently because of the research that they* [with research positions] *have done…*” (M6). Research positions supported projects that led to changes in service delivery models, with another manager commenting, “*…it’s amazed me that through the research grant that she got for that project, she has now generated for the Health Service recurrent money for the full time* [implementation of the] *… rural allied health model*” (M3). Research positions also described helping clinicians build their skills in evidence-based practice to implement research evidence to improve patient care, “*they’ve* [clinicians] *learnt to initiate or support discussions about treatment options with other health professionals…so there are many examples where their actual practice is changing*” (R8).

### Research outputs

Research positions were reported to contribute to an increase in traditional research outputs, including journal publications, national and international conference presentations, and grant funding. A clinician commented, “*… achievements would be the number of* [successful] *grant applications … that we probably wouldn’t have even thought about previously*” (F2). A manager commented that, “*… a lot of staff now are engaged in higher research degrees*” (M7). Supportive mechanisms included the skills and track record research positions brought to the role, “*…having something with a track record …alongside you, obviously it’s easier to get money and you’ve got someone to guide your research question as well*” (M1). Another manager reported that, “*individuals need time in the role as well to get some momentum, get the relationships in the department, get the research programs going*” (M5). It was important for the research positions to balance time spent on their own projects, “*There’s always a bit of a tension… of competing time. How much time do you spend in a developmental sense versus progressing around research?*” (M5). Potential examples to assist in finding extra time included delegating to other supports (M7) and funding to provide administrative and research assistant support (R7, M4). Another mechanism described by a manager included, “*individuals need time in the role as well to get some momentum, get the relationships in the department, get the research programs going and there's usually a delay until you start to see the pure research outputs*” (M5).

### Collaborations

The research positions were reported to have successfully assisted clinicians form research collaborations and networks with a variety of stakeholders. “*They can certainly facilitate people to …find those links and get to the right person. It’s not even the same institution… that sort of pushes people in the right direction*” described one manager (M1).

Collaborations included both opportunistic and strategic partnerships with local and international university academics, other research positions and other healthcare organisations. Practical examples of collaborations with universities included “*facilitating the engagement of honours research students with clinical staff*” (M2), “*encouraging staff to gain academic titles with universities to allow for increased access to university resources*” (R2), and “*assisting clinicians in finding PhD supervisors*” (R1).

In addition, the research positions were described to have helped foster new internal multidisciplinary collaborations, “*It’s actually through them having developed networks …that we’ve been able to engage in research that we may not have had opportunities to do. So medical* [and] *nursing colleagues have thought of us, which is great*” (M7). A common mechanism for facilitating collaborations was the research position’s understanding of two systems, “*How they* [healthcare] *work and how universities think are very different beasts. You do need to understand both of your partners. … I think that to build with external people, you need to think like they do*” (R7).

### Culture changes

Research positions were reported to enhance the culture of research and evidence-based practices across their organisations and teams, “*the biggest thing …for us that we've seen … is perhaps just the culture of EBP and research has been raised in our department*”, described a clinician (F3). Improved attitudes towards research were noted by a clinician, “*…research isn’t this incredibly difficult thing that only very special people can do. Actually, it’s attainable by many and it was quite inspiring actually… I don’t know that that would have been their view prior to this position developing that profile*” (F1). Changes in workplace culture were also reported as a result of this change in research culture. Interviewees reported that their department was seen as a more “*attractive employer*” (M7) and was “*attracting higher calibre staff*” (R8). Clinicians described staying in the health service to undertake research, “*Because these opportunities do exist, these really fabulous clinicians that we have just might stay*” (F1).

### Allied health reputation and profile

Research positions were described to have helped raise the profile and recognition of individuals and teams as active contributors to research, “*So there’s much more of an awareness from the executive and senior management teams that allied health are very active in research*” (R5). Another manager echoed, “*I think it raises our profile as allied health clinicians that we’re in that research space*” (M8). As a result of this increased profile, “*research positions have been invited to speak at organisational events*” (R8) and to “*sit on some of the research committees, that in the past we never would have*” (M4). A key enabling mechanism identified was leadership support, described as “*an incredible facilitator for us. She* [executive director of allied health] *did a lot of promoting us… We could do a lot once we were in the door*” (R8).

### Research infrastructure

Research positions were described to have contributed to the development of advisory committees, “*I’ve set up a research advisory committee. So it’s not just me directing it. I’m kept honest by the committee… I guess that’s part about maintaining sustainability there, that it’s not just one person, it’s a group approach*” (R3). Securing funding for research positions (R3) and developing research strategies were other infrastructure research positions developed, as one research position described, “*… we’ve also gone to departments to help them even develop their own research plans*” (R2). The research positions also developed departmental performance indicators that monitored research outputs and activity, research forums to showcase research activity (M4, M6), and practical resources (e.g. websites, templates) to support clinicians with their research activity (R4, R5).

### Development of individuals in research positions

Individuals in the research positions described their own individual professional benefits from being in the role, “*I think the impact of the role on me has been quite incredible. …how much you learn about the different disciplines and then develop those networks … it’s been a huge learning curve and the enthusiasm that some of the staff approaches their research projects with are truly incredible*.” (R5)﻿. They also described the unique opportunity to influence from within two cultures to make meaningful changes to patient care, “*I couldn't have made that happen in five years …just as a university academic*” (R7).

## Discussion

This project highlighted the potential impact of research positions on building allied health research capacity across health services and described key enabling mechanisms. Research positions were described to have impacted individuals, services, teams and organisations. The influencing mechanisms identified were related to the research position (e.g. accessibility and stability of the role), organisational factors (e.g. leadership and resources) and implementation factors (i.e. how the role should implement strategies). Research positions were perceived as valuable by managers and stakeholders within the organisation for enhancing research, culture and workforce.

Many of the outcomes found in this study are consistent with earlier research, such as documenting increased research outputs, collaborations and research infrastructure [3, 14, 17, 18] as well as influencing clinician research capacity across individual, team and organisation levels [14]. Furthermore, the majority of findings are in alignment with Cooke’s six principles for effective research capacity building initiatives [22]. However, there were also novel findings. These include the outcomes of enhancing workplace culture (e.g. job satisfaction, employer attractiveness) and the reputation and profile of allied health, clinical service improvement, and the professional development of the individual in the research position themselves.

The finding of improved workplace culture is consistent with previous healthcare research that reported a positive association between clinician engagement in research and job satisfaction [23]. The introduction of academic-clinical positions in a neighbouring state was also informally described to help with recruitment of AHP staff [24]. Considering ongoing work retention issues within allied health [25], the finding of improved workplace culture as an outcome of the research positions is meaningful to health organisations. The retention of AHP may be particularly important for rural health settings [24, 26–28].

The reported increases in the internal and external profile of allied health research as a result of the research positions may be also important, given that allied health staff are frequently overlooked at a systems level compared to nursing and medicine [29]. The service and clinical changes described to be influenced by the research positions also provide evidence of the impact of allied health on important organisational performance indicators and therefore help build the allied health workforce’s reputation [29].

Finally, the professional benefits to the individuals in the research positions was also reported in a previous paper describing clinical-academic positions in allied health, stating that the diversity of the roles were both challenging and rewarding to these positions [24]. The stability or consistency of the incumbent in the position was also reported as one of the key mechanisms for their success. It could be argued that the professional development and job satisfaction of the incumbent may be important in promoting the stability of the research position.

Many of the outcomes from the present study also interacted with one another and likely had flow on and synergistic effects, as described by Cooke [22]. Individuals in the research positions described a pattern where the up skilling of individual clinicians led to increased research activity across different allied health professions, which in turn may have contributed towards clinical and service changes. Collectively, these changes and increased research outputs may have increased the profile and research culture of allied health within the organisation. The outcome of improved clinical services and patient care as a result of the positions may be of particular interest to health services and suggests that the use of traditional academic metrics to evaluate impacts of research positions (i.e. publications, grants) may need to be broadened in the healthcare setting in order to capture the true impact of these positions.

The current research also highlighted enabling factors to the reported outcomes of the allied health research positions across different organisational contexts. While a number of unique mechanisms were identified, many have previously been supported in the literature. For example, Perry [17] reported that organisational culture, managerial support and the interpersonal style of the individual in the research position were mechanisms to their success. Implementation factors, including tailoring strategies used by the research positions according to context and readiness, are also in agreement with William et al. [14]. Such individual tailoring is further in line with principles of adult learning [30] and with Roger’s diffusion theory [31], which states that innovations are adopted at different rates according to individual readiness. Furthermore, integrating research positions between the clinical and academic environments, and integrating research into routine clinical activities has been reported as another enabling mechanism to research positions [1, 17]. Other mechanisms related to the accessibility, experience, stability of the research position, physical resources and funding, and other implementation factors are not well cited in the literature and provide new evidence for how the implementation of these positions may be facilitated and supported.

### Key implications and recommendations

The present findings identify a number of potential benefits of research positions across different governmental health services. A variety of mechanisms were identified that have implications to individuals in the research positions, clinicians, team leaders, reporting line managers, university partners and state-wide governing bodies.

Based on the mechanisms identified in the present research, individuals currently in or seeking to commence a research position within a healthcare setting should actively integrate themselves into their respective health setting (e.g. sitting on committees) and maximise their own accessibility and approachability with clinicians. They should seek to utilise existing resources and networks and seize opportunities to showcase research progress and success. Other recommendations include tailoring interventions (i.e. mentoring, training) to clinician’s developmental level and readiness, integrating individual clinicians’ motivations and current research skills, and setting realistic expectations.

Clinicians who have successfully engaged with individuals in the research position and are participating in research should be acknowledged and supported to present their findings, not only at conferences, but also internally to medical and nursing peers. Further, they should be encouraged to support their peers to build research skills and engage in research to build internal capacity, increasing the profile of allied health and developing long-term research sustainability. Operational support for clinicians who are engaging in research is also needed (i.e. finding backfill for clinicians who receive grant funding, support conference leave for presenting clinicians). Clinical leaders should seek opportunities to undertake research themselves, thus serving as a role model within the department for engaging in research.

Interviewees also revealed some enabling mechanisms that reporting line managers of the research positions may consider when managing the research positions. These factors included, where possible, facilitating visibility of the role within and outside the organisation and their research achievements (i.e. publications, support on projects) across different levels and professions (i.e. including medical and nursing peers). When seeking to recruit new or additional positions, managers should first consider mapping the research needs of the allied health workforce within the organisation, defining the purpose of the research position (e.g. research coordination, driving own research agenda, novice capacity building or combination) and then appoint an incumbent that matches accordingly. Managers should also consider the interpersonal skills, and existing networks and experience of the incumbent (including knowledge of internal systems) during recruitment. There should be regular communication with the research position to troubleshoot any operational barriers they may encounter as well as factors that promote the individual in the research position’s job satisfaction, including their professional development opportunities and aforementioned supports.

Certain outcomes of the research positions (e.g. increased PhD students, clinical changes, stronger collaborations) may be appealing to universities when considering a potential investment in conjoint allied health research positions within healthcare settings. Clinicians were able to translate research findings into clinical practice when working in a clinical setting. This can lead to a greater ‘societal impact’ from research, an outcome being recognised more widely as a performance indicator for research institutions [32]. As well as becoming more aware of such benefits, university partners should seek to understand the unique challenges of the clinical environment (in contrast to the academic setting) that may impact on the outcomes of these roles. Finally, state-wide Executive and funding bodies may also wish to consider strategies and infrastructure to best support the implementation of some of the identified mechanisms to maximise outcomes of the research positions.

### Limitations and future research

This study investigated the impact of allied health research positions across multiple health organisations and the mechanisms which influenced these outcomes. In this project, all health organisations were within the same Australian state, and it would be important to substantiate the present findings by further research across other Australian states and nations. Future research should also consider stakeholder perspectives of university, nursing and medical staff. As the majority of informants in the focus group were senior clinicians, future research investigating the perspectives of junior clinicians may also be useful. Although authors implemented processes to enhance reflexivity and rigor, it is possible that interviewees agreed to participate and provided responses that were impacted by the investigating team’s positions and experience and this is acknowledged as a limitation. While the present study has revealed important potential outcomes of the research positions, future experimental research investigating the effectiveness and cost-effectiveness of these positions may also be warranted.

## Conclusions

The present findings highlight the potential value of dedicated allied health research positions within an Australian government healthcare setting. Outcomes of the roles identified included building AHP’s individual and team research skills and activity, increasing collaborations and research outputs, improving research culture and clinical services, and enhancing the profile of allied health within and across organisations. Findings have important implications to a number of stakeholders.

It is recommended that health organisations consider how the research position(s) and key stakeholders within their health service are currently implementing key enabling mechanisms that were identified to enhance the outcomes of the roles. These mechanisms may facilitate the ongoing evaluation, support, outcomes and sustainability of the research positions, as well as provide evidence for the potential need for additional positions and/or resources for these positions. The current findings build upon the existing evidence base and demonstrate the potential value of dedicated research positions and the positive influence they may have on allied health research capacity and culture, clinical services and, ultimately, patient outcomes.

## Additional files

Additional file 1: Interview guide. (DOC 26 kb)
Additional file 2: Additional supporting quotes. (DOC 197 kb)

## Abbreviations

AHP: allied health professional
