# Supplementary material for: Allied health research positions: a qualitative evaluation of their impact
Source: Health Res Policy Syst. 2017 Feb 6;15:6. doi: 10.1186/s12961-016-0166-4 (PMC5292788; doi:10.1186/s12961-016-0166-4)
Supplement: Additional file 1: — Interview guide. (DOC 26 kb) [file 12961_2016_166_MOESM1_ESM.doc]

## Supplementary File: Interview Guide

### HP Research Position interviewees

1. Describe your research interests or background and how long you have been in the present role.
2. Can you describe what you would consider some of your biggest successes in the role have been?

-What factors (within your organisation) contributed to the success?

1. What are some biggest challenges you have encountered as part of your role?
2. What initiatives have you undertaken within the organisation which aimed to facilitate the development of AHPs (Allied Health Professional’s) research skills?

-What factors helped to enable these initiatives?
-What barriers did you encounter?

1. How has your role facilitated AHP research that is close or meaningful to practice?
    -What factors help to enable this?
    -What barriers did you encounter?

How do you think your role has directly impacted on services or client outcomes?

1. What factors have helped to enable you to build research linkages, partnerships and collaborations between AHPs and other stakeholders while in your role?
    -What barriers have you encountered ?
2. What factors have helped to facilitate appropriate dissemination of AHP research findings while in your role?
    -What barriers have you encountered ?
3. How has your role helped to include elements of continuity and sustainability of RCB within your organisation?

-What factors have enabled this?
-What barriers have you encountered in terms of ensuring RCB are sustainable?

1. How has your role contributed towards the development of structures and processes that are set up to enable smooth and effective running of research projects?

-What factors have helped to enable this?
-What barriers did you encounter?

1. Is there anything we have not discussed that you would like to add in regards to your role in building AHP research capacity?

### For stakeholders (reporting line managers and focus group)

1. What has been your level of engagement with the research fellow position(s)
2. What you would consider some of the biggest achievements of these positions have been in terms of building AHPs research capacity within your organisation?
3. What do you think enabled these successes?
4. What do you think some of the barriers are to these positions in terms of building the research capacity of AHPs within your organisation?

*(more specific questions related to specific aspects of research capacity building e,g., development of research skills, impact to clinical practice, dissemination, sustainability, impact on research culture and profile of allied health, may also be discussed)*

1. What factors will facilitate ongoing success of this role?
2. Is there anything we have not discussed that you would like to add in regards to the role of the HP research position in building AHP research capacity?
