# Supplementary material for: Allied health research positions: a qualitative evaluation of their impact
Source: Health Res Policy Syst. 2017 Feb 6;15:6. doi: 10.1186/s12961-016-0166-4 (PMC5292788; doi:10.1186/s12961-016-0166-4)
Supplement: Additional file 2: — Additional supporting quotes. (DOC 197 kb) [file 12961_2016_166_MOESM2_ESM.doc]

# Quotes to support key outcomes and mechanisms

## 1.Clinician Skill Development

| **Examples and/or mechanisms** | **Quotes** |
| --- | --- |
| **Individual mentoring tailored to developmental level** | “… they’ve got a good awareness of where a research student is on their research journey and then is able to facilitate their development.[“F4]  “The other thing that I do is I prefer to do one-to-one mentoring of people rather than the education program, so I do - whenever anybody is interested in doing a research project, most people know I'm around now. I tend to, from the very beginning, sit down with them and help them plan their project and let them go away and do each stage, but I meet with them prior to each stage .”[R4]  “So not just saying, off you go and do that. I spend lots of time and give lots of examples. Spend time structuring their talks, practicing things so that they feel confident and ready and it's a quality thing when they do it” [R7] |
| **Support throughout process** | “(Research position name) was there holding my hand the whole time and being like that's okay, we'll just do that instead. Or that's okay, we'll do this instead. So if it wasn't for her, I don't think I'd be where I'm at with this project because I'd hit a wall and I wouldn't know where to go. But she knows all the avenues and she can be like do this instead, and we did” [F1]  “So she's supported me to be able to apply for research funding, get a couple of research studies up and going, apply for ethics, get publications accepted, been overseas presenting research and I've just got two new studies started up to this year. Yeah, I don't think I could have done that without her. [F4]  “…again at the practical level, we'll help people right through the process. So from the idea, to the grant writing, to the ethics application. So for example in social work, completely helping them right through the process and will now help them actually - and Anna will do that - actually help them write it up to be published. That's been - so helping people write their grants. “[R2] |
| **Breaking down information** | “it 's about just pointing them to the right form, or describing the terminology that an ethics committee has come back with. They're like, what's a protocol? Or what is that version number? What am I talking about? So very basic things. As an internal resource, I think it's really helpful just for getting through the silly questions that you wouldn't otherwise want to ask. “[R3]  “...speak in English and translate research speak into common language” [F2] |
| **Clinician Motivation** | “I find as well that she keeps you motivated. That position keeps you motivated because you're like I feel like I'm still treading water. Speak to {Research position name} and she goes, with her experience, right, you're on the right track. What are you doing next and things? So that kind of does become clear in your head. As someone who doesn't do it all day every day. “[R1] |
| **Empowering self-directed learning** | “Not so much telling you what to do but putting you in the direction and then assisting you if you run into any hiccups…” [F2]  “…the principle of getting people to learn themselves and help them along the way and then they should be able to keep going. So not doing everything for them but using the opportunities of helping them for them to actually learn and then keep going themselves .”[R2] |
| **Availability of Research position**  *Lack of time of Research position* | “I suppose the tack that I've taken is that it's helping others first, so that's very much being there, just be on the end of the phone and people not worrying. You know that whole, oh they're a really busy researcher so I'm not going to bother because they've got to meet - do their own thing? “[R2]  “We just basically were always open door. We're here. Come and pick our brains about anything, at any time. [R7]  “… you do have to get familiarity and some street credibility and all that sort of stuff before they'll come and ask you for help (R1) |
|  | “…apart from one other colleague, there's been no others that have been able to tap into those people due to the fact that (Research position name) is full at the moment and these would be very novice researchers that would need a lot of guidance. So when they come to me, when they get to a point where I can't help them anymore, it's kind of a brick wall which is a real shame at the moment…”[F4] |
| **Approachable/ non-intimidating** | “One of the challenges in fact has been my seniority. That's potentially daunting for others … I think the organisation recognises the benefits but would also indicate that that's been somewhat of a challenge.” [R6]  “Probably very patient. So they're coming to something that's really quite scrappy or in its really basic draft but going, and this is how you can improve it. So probably acceptance of all of our levels of skills and varying levels of that. [F2]  “(Research position) is very approachable and respected and easy to work with. So that's much more encouraging to deal with that type of personality than others. [F1] |
| **Experience and skills of position and research team** | “I would say in all of these positions is, you need people with research experience. I think they're very hard jobs if you don't have some skills and time under your belt. Because you - yeah. You are constantly having to respond to all sorts of different things. [R7]  “so some of them are more qualitative researchers who are happy to play with randomised control trials and vice versa. So they're not stuck in their experience or their expertise area. They're happy to kind of go to whatever the service need is.” [F2]  “We've got a good team, too, because we've got a spread of skills…I never feel worried about anyone approaching us with any sort of research…” [R7] |
| **Physical proximity** | “.. if they're in the same department and you can chat with them in the hallway they're just real people like you and it's easier to communicate with them” [M8]  “.. When I first came here I made sure I had lunch every day in the lunch room and you've got to get familiar for them to - otherwise they won't approach you, they just won't” [ R1]  “Yeah and I think the physical availability I'd vouch for as well. ….. I don't think it would work the same if they were someone who was just based at a university who wasn't based here as well or was just someone that sat up in a different department who wasn't within it. “[F4] |
| **Departmental support** | “We definitely need the support from our line managers to say yes it's okay to not do patients for one afternoon while you go and meet with the research person. “ [F2]  “It does depend on the amount of support that you do get from your line manager I think and the value that they see. I think if you can get that one meeting with that research officer and work out … look, I have to be able to spin this to my line manager that it's going to be worth the time me not seeing patients...” [F2] |
| **Be aware their role involves capacity building** | “So if you're coming to us to want to just do research, then we're probably not the right environment for you. So it was about recruiting the right person, but setting the clear expectation before they came and leadership” [M2]  “I'm always looking for that person who has that clinician focussed - capacity building focus…There’s been excellent, personal-driven researchers, but they're not right for this job…”[R7] |
| **Flow on effect** | “so upskilling - people who are more experienced to then upskill the guys who are thinking about research. So it's a really nice flow on effect. Probably a little bit harder to measure, but I think it is definitely there.” [F2]  “…we don't have a great history of researchers amongst the clinical staff..I suppose, impact with mentoring me through my PhD I've been able to then play an intermediate role …and to helping them design research projects and get things going [F4]  So how I see the benefits was installing the skills, those fundamental skills in someone, can instantly be passed on to someone else if they're shown in the correct way. So that's been a really great impact to give me the confidence to go help others start their journey and also reviewing their practice and things like that [F2] |
| **Example: Team based training and education** | “They also have like dedicated sort of sessions, like tutorial days or video conferences or whatever just on particular topics. So if people don't really want to take the step, they can sort of do it in a - like find out, how it is way first, so that's helpful to some staff “ [F4]  ‘A research skills workshop, and they ran that over a number of weeks and it was made up of lunchbox sessions of where they were covered right from the beginning of what is research to quantitative, qualitative research methods, governance, ethics approval, all the different facets and just did little snapshots over a number of weeks” [M3] |
| **Mechanisms** | |
| **Tailor to needs of the team** | “So it's trying to tailor what people actually need…I suppose tailoring to what individual departments or even individual small groups have…and fitting in with their time.” [R2] |
| ***Consistent time and place*** | “..but part of the success for the 8:00 am group was the fact that it's the same time, same place every week. Whereas with our research education one week it would be here and another week it'll be there so that is difficult.” [R5] |
| ***Keep more experience based*** | “Providing lectures and that sort of thing only captures a certain proportion and there's only a certain amount of retaining. If you really want someone to know something or to experience - you've got to give them an experience of it at a small level and a taste of it and let them see that it's possible and that they're capable of it.”[R1] |
| ***Embed into Professional Development*** | “We would include research as a component of our professional development meetings.” M8 |
| ***Access to existing resources*** | “If you go with a simple question it can be, this is a resource that you can look up and then develop those skills from and that's something you've got lifelong as well. Not necessarily having to sit down for a whole one or two hours with that person.”[F4]  “We have been obviously tapping into the resources that are available. Like I know [another HHS] did an introduction to research design. So a couple of our guys were able to tap into that.” [R3]  “So [university] do a tremendous course on writing, academic writing, which is just absolutely brilliant. So we've got that saved, and I get them to - if they're going to be publishing then I get them to watch that academic writing series. “[R4] |
| ***Inviting external speakers*** | “…we also have done research education series which is now being taken over by our research support unit which I'm very glad about because it's very difficult to juggle all of that .”[R5]  “So [Research position name] actually got the health economics people up to run workshops. We've had this wonderful person from JCU come over and run regular writing workshops.” [M6] |
| ***Barrier of funding*** | “…another barrier is that I don't have money for training. So we have to do this based on - I guess - free will of people...”[R3] |
| ***Clinician time barrier*** | “that seems to cover most of that is time. You need time to do the training. You need time to sit down and think about these things. If you're already - if you're running yourself ragged somewhere else and you've got all patients and all those kinds of things, where are you going to fit the time in?”.[ F2] |

## 2. Increased Research Activity

| **Examples and mechanisms** | **Quotes** |
| --- | --- |
| ***Examples of increased engagement*** | “Just thinking within occupational therapy the number of conference presentations and potential movement into research is probably really driven by that position “[F2]  “But really getting some of those departments that weren't doing anything to actually be doing something is a big success.”[R2]  “I've gone from when I first started I think there was probably less than half a dozen people actively participating in research. I think I've got over 40 research projects currently in process now” [R5] |
| **Mechanisms** |  |
| **Research position factors:**  ***Clinical background*** | **“**…like she's a clinician that is a researcher rather than a researcher who works in the clinical area. So she understands where we're coming from, even if it's not her background, she still can see oh is that how it works.” [F1]  “From my experience having had clinical experience as well, I understood the burdens and the demands of a clinician working in a tertiary hospital, but I also clearly had an agenda and goals to meet from a research output point of view. I think almost all the HP Fellows that were recruited would have had a similar mix of experience and that's important to have that empathy and understanding of a clinical workload.” [R8]  “…to get your foot in the door with the clinicians you need to have a bit of street credibility. Often I think probably if you're a full-time researcher coming in and the clinicians they look at you like, oh -, here's another person going to try to get me to do research whereas if you can run some very clinically based in-services and they start thinking oh, actually they might know what they're talking about here with clinical practice.” [R1] |
| ***Set realistic expectations*** | “Another barrier is incredibly that even though I say from the start when somebody comes to me and says…I want to do some research, I go, okay well kiss at least one evening a week goodnight because you're not going to have that anymore. …I just don't know what more I can do than to tell it straight from the beginning.” [R5]  “… the whole time thing, is not necessarily having an understanding of how long something might take to do or what sort of time frames to set around it which might be something that that position can guide around …”[F2] |
| ***Being full time*** | “The position really does need to be fulltime. I know that sounds like I'm trying advocating for my own job, which I am too, but it gets to the point when you are supporting over 40 research projects where people are scheduling time to see you. I'm booking two and three weeks in advance because with two and a half days a week you just don't have enough time.” [R5] |
| ***Respect clinician readiness*** | “I think the important thing to understand too is that there's a proportion of clinicians who just don't want anything to do with research and that's absolutely 100 per cent fine. It doesn't mean they're not good clinicians. ….. The worst thing you can do is force those people to try it because they will leave and they'll go somewhere else and work where they're not going to be forced to do it or they'll reluctantly enter a research project where they'll just - it will be a disaster.” [R1]  “But my take has always been to not to - if they don't want to play then we're not going to - we're actually spending our energy where people do want to play, but it's probably really only been one department that hasn't engaged at all.” [R2] |
| ***Presenting clinicians as role models*** | “But once you see people doing it, you sort of think oh I'm the only one not doing something so I better. “[F4]  “…we particularly picked out some of our clinicians researchers to give presentations on not only what they've done but how they'd incorporated research into their clinical practice. Are they new students to get to undertake research? Even just their research journey. Those talks are really valued by the clinicians seeing their fellow clinicians provide that example and no one can even say here in dietetics there's been lots of - we have staff as the great role models of the - a clinician that just goes through that process. Then they can see that they can do it to. “ [R2]  “With respect to encouraging clinicians to do work in the early stages, we did a lot of - and still do - free talks and they get to see other clinicians doing cool projects which makes them think, oh I can do that too… seeing people fitting everyday research into their everyday work and seeing it as something that's achievable and fun.” [R7] |
| ***Interpersonal skills*** | “So you can have all those characteristics but not have the personal, interpersonal skills to be able to engage with clinicians and to understand the clinician's perspective. So the three of them are all very, very good at that kind of engagement with people. So they're the right people.” [M7] |
| ***Perceived stability of position*** | “So if they think that you're you know, your fellowship is going to end and you'll be somewhere else, they don't want to start things. They want to know that the fellow is going to be around. Whether it's you personally or a fellow, but someone will be around..”[R8] |
| **Attend planning days** | “Attending planning days is one strategy and I know that worked with speech pathology… that's when they became research active, just after that planning day basically.” [M6] |
| **Clinician factors**  ***Clinician barrier time***  ***Clinician persistence*** | “..we wouldn't have been able to do what we do without them really. We can't do this on our own because we're all holding clinical caseloads or other positions, that we've still got a job to do at the same time and even though we all give up time and evenings and at weekends and stuff like that, they've made it possible to be able to achieve these things that we have.” [F2]  “We've had - most clinicians will say I'd love to do some but gee, within my work hours I don't have the capacity. I think that is a genuine challenge and what sometimes promised by the organisation isn't necessarily delivered to the clinician. With the short - how can they be, they have staff cuttages all the time. Their FTE numbers are down. Of course the clinicians are going to be much busier than what they might theoretically be.” [R1]  “that lack of formal research time embedded into clinical roles can be a challenge. I really think that that needs to be improved.” [R8] |
|  | “those that are dedicated to research are the ones that will stay and continue to persevere.” [R3]  “you don't get it to work without a lot of work behind it. But it has been paid off and been well received and that.” [R7] |
| **Project factors**  ***Clinically meaningful projects***  ***Start small***  ***Make it doable***  ***Being involved in experienced team*** | “For clinicians, a lot of the research they look at they think it so lacks clinical relevance that I wouldn't be bothered doing anything like that. Well they can do something that's directly clinically relevant … because it's based on the patients they see. I think that is more attractive to them sometimes.” [R1]  “So when I go down into any department I get them to tell me - what is your service? Where are your problems? Brainstorming their problems… It works in multiple ways in that it's engaging for them, it gives them an answer, but it also enables them to do research in their clinical day. “[R7]  “… the people there at the coalface want to be participating in research that they feel makes a difference to the patient. So you need to get that good balance with all of the multidisciplinary staff that you work with, that they all think that the work that you are doing is important translational work. “[R8] |
|  | “…just letting people know, that the research doesn't necessarily have to be large scale. You know, if clinically you identify a gap or a clinical question or those type of questions, you can do again, small scale things “ [F1] |
|  | “It's making it as easy for them as it possibly can be and that's essential. It's making it clear to them that you're trying to answer something that's going to help them in the long run” [R1] |
|  | “So they're part of the research team and they may not be driving it but they're certainly contributing in some way. So that then allows them to get exposure to how a research program works. What are the pitfalls, what are the disasters, what are the solutions, just getting an understanding about how a research program might run. “ [R8 |
| **Service factors**  ***Leadership support***  **Research as team KPI or part of core business**  **Physical resources**  **Protected time off** | “If you don't have that overall support from a leader, then it just sits and no one does it...”[F3]  “..they'll say we're not allowed to do research because they're told they're not allowed to do research. Because they're told if they are to do it, then they will do it. So it's all about the leadership and then the modelling behaviour.” [R2]  “You can say you're supportive of it but then if your actions don't back that up then that's obviously being seen as not supportive. So, yeah, where possible - it's not always possible but where possible - it might be leading a person in a particular area so they need to work on the research rather than rotating or, again, where possible if they're able to present their research somewhere” [M8] |
|  | “Like signing here at (hospital name) like first up as a new grad, you'd be like okay, so KPIs for the department, 30 publications a year. Like several hundred thousand dollars in research funding, like all of these things and to me, I'm just like oh my gosh, but we do it. “[F4]  **“**But then some departments are now recognising that and are allocating one or two hours a week to research, which is a start. It's not much but it's a start.” [M6] |
|  | “… even just to do a literature review offline. You'd have to go to the library and it's like on the other side. It's just those little physical barriers..” [R3]  “A critical challenge is the access to research, specific software and IT support. …Things like getting access to SPSS statistical analysis which took six months to get uploaded onto the computer and EndNote, getting EndNote to work on our computers. It's a systematic, well systemic issue in terms of the computer, the hardware abilities to cope with a lot of that…”. [R8] |
|  | “So if there isn't backfill or dedicated positions, I think it's (research) much less appealing.”[F1]  “Funding, you know getting grants, funding and all of that has enabled us quite a bit, you know so then staff can be allocated the time or take the time off to do research has been great..”[F4} |

## 3. Clinical and Service Changes

| **Examples and Mechanisms** | **Quotes** |
| --- | --- |
| **Examples** | |
| **Client outcomes** | “That might be initiatives to prevent falls or reduce the number of falls in hospitalised adults, for example, and yeah to then see strategies that have come out of their research then implemented … an example of improving patient outcomes.” [F4]  “But with their treatment of prostate cancer they've actually changed their clinical procedures and they're doing it very differently because of the research that they have done then.” [M6] |
| **Increased clinical staffing** | “One of the key pointers is being able to roll out the research that says well actually, this model is actually really cost-effective when compared to usual care. So that opens doors then to have that service grow…. our screening clinic has basically, in effect, grown in the last 18 months... It was two FTE, now it's up to six…So that's probably a direct clinical impact of that position among other people who are in that position [ M1]  “…it's amazed me that through the research grant that she got for that project, she has now generated for the HHS recurrent money for the FTE that are doing that rural allied health model.” [M3] |
| **Service delivery changes** | “So we've benefited a lot from the models of care projects…but we wouldn't have benefited as much if we didn't have a HP research fellow side-by-side with us helping formulate the evaluation - methodology.” [M2]  “It was my idea. I did the testing. I did the rollout. We did the clinical trials. Now we've got the implementation and economic analysis and now - and it's happening in 20 clinics - or 19 clinics. You just go, that is cool, in five years. I couldn't have made that happen in five years, being disconnected from Health and being just as a university academic.”[R7]  “She’s [research position] done some other research in other areas I know in the community based rehab team where she did a whole research project on skill sharing down there between the nurses and the allied health staff, which created efficiencies in the way they work.”[M3] |
| **Increased implementation of evidence in practice** | “I think overall the greatest impact is having a workforce that understands evidence based practice. If you have a workforce that understands the data that's coming out of the literature, that can read a paper and know what it means and know what that might mean to their practice… then being able to and confident to change their practice. Otherwise, you just have a workface that just keeps doing the same thing.”[R8]  “These are things - these sorts of outcomes where you've got over 80 per cent of the staff now will search the literature to help support their practice. They will use their skills that they've learnt to initiate or support discussions about treatment options with other health professionals…if they're on a (doctors) round and the doctors request something, that they actually have confidence to be able to have a conversation about that decision. [R8] |
| **Mechanisms** | |
| **Actively encourage EBP**  **Projects close to practice**  **Understand the health service context**  **Barriers of translation** | **“**..so trying to get people to think about the evidence and how they implement that in their practice. She [research position] has a competition around that.” [M8]  “… getting people to see just in their day to day practice whether it's something that they've directly discussed with the research practitioners, am I really - is this really the right thing to do? Why am I doing this?” [M7] |
|  | “So we do - very much, with the early first projects - try to make them absolutely as close to practice as possible. Because that is the most chance that it's going to be successful. Because they can do more in their day relating to it, they know and love it, they are often the expert in it…” [R7]  “I think that was the philosophy is that we wanted it to be stuff that would make a difference at a clinical level and really having the clinicians drive the research questions and the research thing from there.” [M8] |
|  | “she's able to apply it back to the health service here because she's got that understanding of it.” [F1]  “(Research position name) had the clinical background and she intimately knew our clinical business…when a couple of things happened over the last 12 months we got a huge investments into allied health…(they) just jumped into the middle of that and immediately initiated a research framework around that. [M5]  “…that local knowledge and local connection helps remarkably. That allowed me to already have contacts within departments who knew me, trusted me and had worked with me previously. … when you are looking to change practice, those things have to be earnt and built up. “[R8 |
|  | “ there's just those sort of normal - all the normal translational barriers. So just the economics. So just how to smooth that over - and none of that is ever going to be easy. Otherwise everything would always be automatically adopted. ..”. [R7] |

## 4. Research Outputs

| **Examples and mechanisms** | **Quotes** |
| --- | --- |
| **Examples** | |
| **Publications and presentations** | “Just thinking within occupational therapy the number of conference presentations and potential movement into research is probably really driven by that position” [F2]  “The position holder, who's been in that position several years now or quite a few years now, is publishing half a dozen plus papers every year. Very very productive position.” [M5]  “I mean obviously we have a lot of success in terms of your typical or traditional metrics, such as publications and funding and all that type of thing.” [R8] |
| **Grant funding** | “I think some of the other achievements would be number of grant applications and number of applications successful out of that, that we probably wouldn't have even thought about previously.” [F2]  “..first of all, it's been the amount of grant funding that I've been involved in or directly attracted, which is more than - it's about $4 million, either as a co-investigator or a primary investigator. So I think that's been quite a success.” [R4]  “We've had enormous success by way of grants which would be in the vicinity of a million dollars…” [M5] |
| **Research Higher Degree Students** | “So I'm currently enrolled in the masters by research and looking to articulate to a PhD in the next couple of months and briefly without (Research position)’s support I wouldn't be enrolled in a high research degree and had success last year in achieving a HP grant so he's been pivotal in that.” [F4]  “I think the outcome is that they gently entice clinicians into undertaking research and engaging in higher research degrees. I haven't counted them but we have a lot of staff now who are engaged in higher research. “[M7]  “I guess the measurable things that we can take out are the numbers of PhD students has increased and we have I think it's about five or six PhD students that are Allied Health professionals now that are clinicians here. We have one recent graduate who's just finished her PhD.” [R5] |
| **Mechanisms** | |
| **General mechanisms for promoting research productivity**  ***Find balance between supporting clinicians and output*** | “Some people are just terrific researchers and can just output papers. Dozens every year. But they're not necessarily the right people to support novice researchers. Sometimes they are but not always. The same if some people enjoy the mentoring and the support of novice researchers and that can become very time consuming and a good investment in that space can really then limit what you can produce yourself.” [M5]  “[if] you just focussed on what you get the clinicians doing and research building capacity you wouldn't have a lot of output or it would take you a decade.” [R1]  “There's always a bit of a tension, or challenge of competing time. How much time do you spend in a developmental sense versus progressing around research? It's a bit like the clinician having a clinical load and then trying to find time to do the other things. [R6] |
| ***Extra assistance and support*** | “…we're a research team, but we have no administrative support or anything like that. So I'm the one fixing the light bulbs...”[R7]  “The research Professor has had a research assistant with some part funding that we've been able to achieve… they'll often ring him first, and (assistant name) will go down to them and talk to them about something…. I think that has certainly helped. [M4] |
| ***Have research strategy*** | “…build a research program and get funding for a research program and then bring Allied Health clinicians offline to help with that research and be involved in that and be involved in publications and then hopefully go off and do research themselves, or support PhD or Masters students”. [R4]  “Yeah, generally it's part of the project management of any sort of work that we're doing, we would be planning the manuscripts and the conferences at the beginning and thinking about that strategically where we'd be targeting that dissemination.” [R8] |
| ***Being full time*** | “I think the other challenges for [Research position name] is because it's part-time it's getting her research done as well. So that's been a real challenge and she has taken it on board now, so she's learning to do catch-up writing and so forth to try and keep up her publications, which is essential for her position.” [M6] |
| ***Encourage and support clinicians to disseminate*** | “...and making sure that I've got as many opportunities for people to present at. Then you make it fun, whenever you're there. It's a social event. It's a good thing and they get a buzz out of it and then you get them to talk to other people about why it was worthwhile. Because everyone gets a buzz out of presenting your stuff and people thinking it's cool.” [R7]  “…assisting in the writing process is a really important role of the fellow to assist the clinicians to get it across the line. The reality is, it isn't until it's published, it doesn't exist. So making the work exist is what I see as one of my crucial roles in supporting staff to write, teaching them how to write in a scientific way and really keeping that encouragement going because it can be a hard road. But that's really important to disseminate that information and helping staff to also strategically choose where to target, where to send things, so that's where I guess my experience comes in.” [R8]  “…when they [clinicians] will not use a second of their own time - that is a barrier. But legally they're not supposed to or have to, but if they just won't stay the extra half hour then, yeah, everything just slows down. But it's just been ways to rethink that and personally, myself, learning to go at that speed.”[R7] |
| ***Access internal publishing team*** | “…the best thing is that we have a fantastic publishing team who are actually willing to assist people with presenting their posters better, and working with them on their presentations, et cetera. So that has been a blessing... “ [R3] |
| ***Time and stability of position*** | “The positions need some time in place but individuals need time in the role as well to get some momentum, get the relationships in the department, get the research programs going and there's usually a delay until you start to see the pure research outputs.” [M5]  “Stability of the person in the position is really important, because if you bring a new person in, it takes - because we've had some research positions change over. It takes a couple of years, realistically, before they've ramped up their productivity. “[M1]  “... so you really need to make sure that you have it [research position] at a reasonable level, so that you can actually get some length of time, some - to get runs on the board.” [F3] |
| ***Management support*** | “They can achieve a lot more if the manager is supportive. We had key performance indicators around research that they exceeded every year. I think having the rest of the team leaders who research wasn't their number one thing they wanted to do in life but they were supportive.”[M8] |
| **Mechanisms for attracting RHDs**  ***Present clinicians experiences***  ***Embed support within organisation***  ***Barrier of clinician’s reluctance to give up role***  ***Being linked to university***  ***Choosing right project topic*** | “ I would easily say, probably three people I've now got enrolled at PhDs came out of seeing that sort of talk.” [R7] |
|  | “I think it was about $15,000 or $20,000 each which allowed somebody offline a day a week for six months. The whole idea of that was to try and enrol them [clinicians]. These were people that had a goal that was achievable within 12 months of enrolling in a Masters or a PhD and they had a map of how they were going to get there. It was sort of like a pre research higher degree scholarship to get them in.” [M5] |
|  | “…there's that real sort of undercurrent of not wanting to give up their role.. So they (clinicians) sort of feel that ooh, I have to give up my job and go and do a PhD for three years and not get paid much money…I find that a challenge to actually convert in house research activity into a higher degree for staff…” [R8] |
|  | “… you need to be linked with an enrolling institute to be able to be a supervisor. So they're really - is a critical aspect to having those links with a university, to be able to manage that sort of stuff.” [R8] |
|  | “I suppose, even when a clinician comes to me now and says I want to do a PhD - I work with them to design a project, still in that same way. Like, I go, oh, who are you seeing every day? Who's in your clinic? Not the, let's take you out of that and let's do this question. You might want to do that question, but you're never going to do it as a full time clinician.” [R7] |

| **Mechanisms for attracting funding**  ***Track record of research position***  ***Encourage clinicians with grant applications***  ***Supportive department to manage funds*** | “…having something with a track record, such as that position, to be alongside you, obviously it's easier to get money and you've got someone to guide your research question as well. So those are the big benefits.” [M1] |
| --- | --- |
|  | “So HP grants are due next week. Basically they all need to go to [Research position names] for them to actually have a look at them to help improve, if necessary, before they're signed off by the ED of allied health. I mean that's that primary process, but yeah, it's saying that there's this practical help there.” [R2]  “she's really promoted that and I think that's made a huge difference. Because otherwise I'd have no idea what research grants are around “[F1] |
|  | “I think for me the most valuable thing is a supportive department who can sort your financial stuff out without you having to know all that.”[R1] |

## 5. Collaborations

| **Examples and Mechanisms** | **Quotes** |
| --- | --- |
| **Examples** | |
| ***Create or strengthen networks with University partners***  *Use of honours students to do research* | “We've got the patients and the data but if we don't have the researchers and they're sitting in the uni, she can be the conduit between - other than just developing researchers in the hospital. “[F2]  “They [Research position] can certainly facilitate people to do - find the right person, if you will, find those links and get to the right person. It's not even the same institution, it's at Australian Catholic University, but that sort of pushes people in the right direction.” [M1]  “…So I felt like I had avenues to give other people, because I knew the strength of UQ and I know what UQ can do. [R7]  “The other thing that role has been instrumental - the research fellow role - in securing an agreement with the University of Southern Queensland for having some student placements here in their honours year. “[M2]  “I also have negotiated with our two primary partners that we take students that we're then a preferred partner to take the research student - honour students and negotiated that we get a proportion based on the number of clinical students that we take. So we get a guaranteed number of research honour students each year for example.” [R2] |
| ***International collaborations*** | “The project that I put in for with [Research position name] is something that hasn't even been trialled in Australia yet. We've just worked really hard together on it and my main associate investigator is over in Canada and I would have no idea how to talk to him about it and how to say this is what we want to achieve in this setting, how did you do it over there? …But she helped me word it and she helped me say it and we've got in contact with him and he's on board.” [F1] |
| ***Third parties*** | “So our particular research fellow actually worked with our Medicare Local. So there were links there as well with the Medicare Local… So she developed those links, so there was research conducted with Medicare Local.” [M6] |
| ***Internal collaborations***  *Within allied health* | “I think something [Research position] has also really cultivated in our cancer areas is a lot of cross disciplinary research. So we've got a lot of projects where there's OT speech, radiation and dietetics and we're all working across different projects which again I don't think without her position there...”[F4] |
| *Medicine* | “So there are different aspects of research that she has brought forward and collaboratively work again working with medical mostly, having that breakthrough and creating sort of the opportunity that we can contribute in medical research as well has been really great from that point of view.” [F4]  “..it's actually through them having developed networks meant that we've been able to engage in research that we may not have had opportunities to do. So medical nursing colleagues have thought of us. Which is great .”[M7]  “… we've had strong medical research happening. Probably the way in which she (research position) has helped us to include allied health in those multidisciplinary projects.”[M6] |
| *Health managers* | “…it's the health managers and the people who can make things happen. ..and they're people that you've got to get on board with things because they can make things happen from a service perspective. It's also how to give a lot of advice about who you want to collaborate with as well.” [R1] |
| **Mechanisms** | |
| ***Time in positions*** | “You have to be very invested in the departments and have that buy in, and that takes years. ...I think sometimes people come in thinking, okay, so I'm the researcher here now, and they're all just going to want to do stuff with me. It's like, that's not how it happens. This takes years. Years to build the relationships, years to build the trust…” [R7]  “It takes a little while to build networks and build up.” [M1] |
| ***Experience and existing networks*** | “HP fellowships are more around trying to get some experienced researchers in who know a lot of people who can help out if the project that's being talked about is not their area of expertise.” [R1]  “I suppose because of the fact that I came with a long history at the university - so that was a true strength. So I know that department well, I know everybody well. Then, nationally and internationally, I know a lot of people. So my experience was a big facilitator with building external collaborators, because I already have a lot of collaborations with people.” [R7]  “..so someone like myself where I already had a track record and I already had research networks, and I was able to hit the ground running in terms of my own science was really important because I wasn't starting from scratch.” [R8] |
| ***Conjoint position*** | “…that particular one (research position) is a conjoint position with UQ, so that gives us a really good link with the University of Queensland.” [M1] |
| ***Understanding strengths of both partners*** | “I think the understanding that the clinicians do have a very good, very diverse set of skills and the researchers have a very different set of skills that if you do get them together talking nicely and collaborating with a respectful understanding that that can work well. “[R1]  “I'm lucky, because I have had so many years of history dealing with - so I know the university world. So I know what they care about. I think some people who have only ever worked in research positions here in health - how they work and how universities think are very different beasts. You do need to understand both of your partners. … I think that to build with external people, you need to think like they do.”[R7] |
| ***Knowledge of helpful collaborators*** | “Because at the end of the day, research is all about relationships and it's who you can work with. If you can't work with someone, you can't research with them. So if lots of people know you and trust you - if you say, look, this person is good, work with them, and vice versa, that really makes that external collaboration stronger.” [R7]  “One of the things you get to know in universities is who is an expert in what and who you'd collaborate with and who's going to be helpful for people. It's not always the most famous one.” [R1] |
| ***Co-supervising students across specialities*** | “So I might co-supervise a gastroenterologist or hepatologist or you know, by doing research higher degrees. That's a way then for me to expand my networks and collaborations with the other supervisors and groups that they're working with so that's really important” [R8] |
| ***Physical space*** | “I think their physical set up also has helped their success. Like they've got some office space, places where you can go and meet one on one or have a collaborative discussion with more people that's required and it just appears that they've physically got the structure to enable that research to happen. It's not trying to find a room or desk space each time you need to meet. There's normally a good spot.”[F4] |

## 6. Cultural changes

| **Examples and mechanisms** | **Quotes** |
| --- | --- |
| **Examples** | |
| **Research culture changes** | “Well probably the biggest thing in occupational therapy for us that we've seen in connection with [Research position name] starting I guess is perhaps just the culture of EBP and research has been raised in our department...” [F3]  “I think over the last two years there's a significant amount of work being done in a couple of those other departments. Mainly social work and OT. I think there's no question that the culture has changed.” [M5]  “Which has been quite a culture shift from having research as an added extra or a luxury or it's something that only certain people get picked to do, to now being I guess an environment where everyone that comes to work here expects to be able to participate in some way in research. So I think that is definitely a success change in culture, in my view is a success. [R8] |
| ***Research discussed more*** | “..if I think back to a year ago when we'd have our …team meetings and you'd have your agenda items, research was never on it. Now it is.” [F1].  “I'd just say it's [Research] being discussed more. It's a word that is in conversations.” [F1] |
| ***Attitude changes*** | “To me, it seemed like going that way really created this sense of research isn't this incredibly difficult thing that only very special people can do. Actually, it's attainable by many and it was quite inspiring actually. It's something - if I think about the people in the team that I lead, I think they'd all pretty much see it as possible. As something that could be on their horizon. I don't know that that would have been their view prior to this position developing that profile.” [F1]  “Yeah, it's [research] not the elephant in the room. It's not the scary thing [unclear] imagine a lot of them might think” [F2] |
| ***Flow on effect*** | “…people who are more experienced to then upskill the guys who are thinking about research. So it's a really nice flow on effect”. [F2] |
| ***Workplace culture changes*** | “…the biggest achievement I would say, you know, having the research fellow in there and ongoing research promotion from the departments the more attracting high level or high achievers staff coming in as well and applies to come and work for as opposed to about five, six years ago when we didn't have that sort of rapport.” [F4]  “But also changing the culture within the department to be more of a research focused culture which I think then is actually one of the reasons people want to come to work at [hospital name here] is because of the research culture. [M8]  “Because these opportunities do exist, these really particularly fabulous clinicians that we have just might stay in this little dinky regional health service rather than take off to the big smoke. “[F1] |
| **Mechanisms** | |
| *Up skilling and increased confidence* | “I think the culture change is probably about the upskilling and people's increased confidence and [the] increased recognition of the value of that and the sense that what we have been doing can contribute more broadly outside of the HHS into specialty areas ...” [F2]  “Some teams have definitely changed. I think those teams are the ones - probably the smaller ones that didn't really have the skills or the access to universities to assist them to develop the skills. So they have been enabled through this role and have taken it on board and are now research active…”[M6]  “Yeah, so I suppose all the people I mentor - is, I suppose, giving them the confidence that they can do that now. Because some people go, oh, I still couldn't do it myself. It's like, of course you can. So I suppose it is a bit of that counselling of the clinicians we mentor, that they, too, can - they can do it.” [R7] |
| *Existing research culture* | “The hospital itself…is very much supportive of research. There's already a culture of research within the hospital which may not be as strong as some of the other ones” [M8] |
| *Leadership role modelling* | “So if your leadership model is that research is part of what we do and we need to build it into our everyday work and all those sorts of things, then the staff will follow.”[R2]  “I think probably at the time had a new director quite a view years back who had a personal interest and I think led by example. So was doing some research herself and so everyone thought, oh look if you can do it as a director, with all how busy you are, and just I think that and basically it was just like an expectation pretty much. “ [F4] |
| *Communicating research updates to Executive* | “So some of that culture still obeys, but I think because of the way we're working right across allied health and discussing - so doing research updates at all of our Executive meetings, really talking about the culture more and it's [change is] happening.” [R2] |
| *Seeing research as something that can be attainable* | “Because I didn't do research as part of my university degree and I very much regret that. But just going through with [Research position name], she really dumbed it down to my level which is great. Now I feel really confident in doing it. I think this is only the start for me in research. She's really inspired me which is good.” [F1]  “She really built a profile and a presence I think and brought in various colleagues to run workshops and qualitative and quantitative and how to write various aspects of research and so on. To me, it seemed like going that way really created this sense of research isn't this incredibly difficult thing that only very special people can do. [F1]  “I think that's similarly like with social work generally, we started like a clinician initiated research group and [Research position name] came in on that and I guess made the idea of research not to be such a big thing. [F4] |

## 7. Allied Health Reputation and Profile

| **Examples and mechanisms** | **Quotes** |
| --- | --- |
| **Examples of profile building** | “I think it raises our profile as allied health clinicians, that we're in that research space..” [M8]  “I think they've been highly valuable in casting some good focus onto allied health research and being able to bring that together in a bit of a coordinated state wide way as well has helped…” [M5]  “the fact that we've had a [Research team listed here] has been important for new staff joining our organisation and for the organisation as a whole to understand and to acknowledge that we are very much engaged in clinical research within the hospital. So it has allowed I think at a strategic level for us to have recognition of the role of allied health professionals [M7]  “…publishing research that has put the department into the forefront” [F4] |
| ***Medical peers*** | “I think it's lovely now that we are starting to get that recognition where you will hear people in medical fields or other colleagues saying, oh yes, there's research happening in nursing and a huge amount of research happening at Allied Health. That's a lot to have them become very aware, because it's been very medical focussed” [R7] |
| ***Senior Management level*** | “..what is perceived as the biggest success at a higher sort of helicopter level, is that that hasn't gone unnoticed within the health service. So there's much more of an awareness from the executive and senior management teams that Allied Health are very active in research….. So there is a great awareness from management level that Allied Health are active in research and I think that that's probably the measure of all success.” [R5]  “So we surpassed nursing considerably attendance-wise [at HHS conference], abstract-wise and we were right up there with medicine and so that was recognised by Exec and research units. So it was really a huge benefit for our research fellow to show her work basically. “[M6] |
| ***National level*** | “I suppose a great sense of pride when I am down at national things - such as a XX conference - and repeatedly having people saying, oh my God, there is so much happening in Queensland. Oh my -, you are involved in so much. There's just so much happening up there, how do you - so I feel that all the hard work is actually - it has visibility and people have noticed it, which is fabulous” [R7] |
| ***International level*** | “I mean the first thing I said was our international reputation and that's recognised by our invited speaker presentations. I just looked up our KPIs to have a look and over the last three years we've had 16 invited speaker presentations. So that's where either myself or other fellows within the department have been invited to speak as a plenary speaker at an international conference. “[R8] |
| **Mechanisms** | |
| ***Promoting role at presentations***  ***Barrier- policy barriers to travel*** | “I acknowledge my role in Queensland Health. I have this amazing opportunity - involved in this team. It's very unique and we're capacity building…. … I did talks at the beginning about their job. I did a lot of presentations at - like forums, within the university - outside. I did a keynote in New Zealand about capacity building positions and what they can do [R7]  “that position regularly does presentations within the hospital, at symposiums and things like that. Like the one we had this year, {RF name] gave one of the addresses at the previous symposium. So it brings profile to our department as well as recognition.”[M1] |
|  | “I think it creates a barrier when these positions … have to apply for ministerial approval to attend conferences and have their approval in eight weeks out and not know if they're actually approved. I see that as being quite significant.” [M7]  “So the dissemination of research data can have barriers put up, mainly because of the government culture … So there's that real disconnect in terms of the ability to facilitate a lot of travel of staff. Not so much that we get a lot of people who are denied that, but the amount of time and effort and paperwork and bureaucracy that has to occur in order to make it happen. Then a lot of staff are ending up taking personal leave, taking holiday leave and having to do this sort of stuff on their own holidays because it's just all too hard. So that is a shame that that stuff still happens a lot in the hospital systems at least [R8] |
| ***Reputation and experience of incumbent in Research Position*** | “I think the fact that there is a researcher who is quite a prolific publisher, and who is sitting at the table with some of the senior researchers, it does help to - for say the [Research group name], to recognise research has some - a space that allied health does play and just doesn't tinker on the edges of. So I think it has absolutely helped in terms of the profile and the expectation that allied health is part of the planning of research, consideration for research, those sorts of things.{M4}  “She's an international researcher. So we get the kudos of that. I don't think it comes with the position. That belongs to her. ...”[F1] |
| ***Integrate into HHS level Research*** | “..recognition at the ethics committee level of allied health research and any other meetings that she was previously on, so just basically teaching …other people about what allied health research can do. So we do have a good reputation here now at the hospital .” [M6]  “We sit on some of the research committees, that in the past we never would have.” [M4] |
| ***Reporting of research outcomes*** | “…a quarterly research report that's tabled at the Executive. Now that's very powerful for the Executive to see the work that was going on so it's very visible and it keeps - at the level of engagement they can see the trending up, more people involved, the strategies that we identified in the research strategy strategic plan are starting to get some traction. So I think that is an enabler, making it visible but in a formal professional reporting way.” [M2]  “..making sure that every publication that person does or every chapter they write or every presentation they do…gets filtered through to us so we can put it in our research reports, so we can announce it to the world and tell everyone how we're doing.” [M1]  “I mean, at the end of this year, we'll have to have a research annual report. That will obviously go to the board, but it will also be disseminated internally, and that there'll be fact sheets that will be sent out. Posters in the foyer. Just action recognition that a lot of the stuff that's done - you know, in research.” [R3] |
| ***Engage with Executive*** | “.. they (research positions) report to the Exec and stuff like that, so that enables our little projects to get a little bit of a spot like with the Chief Executive and the board and things like that. It just lets them know that we are doing things. Probably then gives us more credibility as well with what we're doing; that we've actually got that research person there.” [F2]  “I think my contribution to success in research here hasn't so much been able to be quantified in helping individuals per say but leading the allied health team…I represent them on all of the senior research committees at the institute for example. As part of that process the education and training that we have proposed in the allied health team has actually led in many cases or contributed to the education and training activities around EBP and research of the wider organisation and not limited to allied health. “[R6]  “I think having the Professor there too enabled that position to be recognised. If we hadn't had the professor there I don't think that position would have been recognised by the Exec. “[M6] |
| ***HHS that values research*** | “…So there's a forum to actually talk about your ideas and get your research known and publicised. There is support within the organisation for that. “[M1]  “I think a fundamental cultural support of research. If you're organisation doesn't have that research positions and research resources can be vulnerable. I don't expect that that will be the case here.” [M5] |
| ***Quality and Credibility of Research***  *Deeper mechanisms for quality research* | “Having those positions when you go back to your team report and you can say that you're doing this in conjunction with this official person who's - that's their title - it gives these staff that don't think - well what are they doing that for, it's just a waste of time - it sort of adds a little bit more credence to what you're doing.” [F2]  “I think really we've been able to grow and sustain a real international reputation for high quality .. research.” [R8]  “So for things like any sort of presentation or talk or poster or anything that goes out of this department, as a research output, it gets reviewed internally first. So nothing goes out that hasn't been seen by a research fellow. “[R8)  “You are not doing a half wrecked analysis of something and getting up there not knowing it. I can't do that, either. So it's just making sure that we have a good plan, that we are well and truly advance planning for things so that we're ready with good quality stuff.” [R7]  “…we also need some kind of structure in place in terms of an admin person so we can implement things like clinical audit of trials, different aspects of trials and randomly check through our governance mechanisms that they're adhering to standards….” [R4] |

## 8. Research Infrastructure

| **Examples and/or Mechanisms** | **Quotes** |
| --- | --- |
| **Research groups or committees**  ***Hindering mechanism - lack of outputs in meeting*** | `  “..set up a weekly meeting for just anybody interested in research and so from that meeting she developed a lot of interest just from the handful of people coming and providing support with them on a weekly basis.” [M6]  “I've (research position) set up a research advisory committee. So it's not just me directing it.… I guess that's part about maintaining sustainability there, that it's not just one person, it's a group approach. [R3]  “We had a [hospital name] research network that we'd developed, which was everybody's name who had an interest in research, so we've mapped everybody who is interested and who has got expertise in research.” [R4] |
|  | “kind of attending, attending, attending with no outcomes at all from that meeting.”[F3]  “I'm not sure that actually being on the committee progresses anything.” [R8] |
| **Securing of additional research support positions** | “The rapport that [Research position] has and her ability to work with other medical and other Allied Health … saw that other research fellows wanted to work under her supervision and under her guidance. So as a result for the last, whatever five years or so, we had at three other research fellows who wanted to come and work in our department.” [F4]  “…one of the achievements was being able to secure some funding to get a research support officer position.” [R3] |
| **Development of research plans and strategy** | “After meeting with everybody I did up a quick sort of operational plan of what we intended to do for going forward which was - it's about doing those - it was the practical stuff but also the strategic stuff… ..we've also gone to departments to help them even develop their own research plans.” [R2]  “.. so we developed a strategic plan around research and EBP. Then…operationalised that through an operational plan. Then we assisted each of the departments, actually supported them in terms of - from their different starting points, their own local support structures and mapping of activity.” [R6] |
| **Integrating research KPIs and reporting**  ***Mechanism- engage leaders*** | “Then I also developed KPI specifically for research. A little bit more detailed than just publications like things like number of grants that actually go in whether they're actually successful or not. The number of staff actually engaged and hours of training that they attend” [R2}  “… anything that we measure as a KPI is put into that tracking document. So that includes articles, manuscripts that are under review or accepted or currently being drafted. [R8]  “…at the end of this year, we'll have to have a research annual report”. [R3] |
|  | “I set this range of KPI which I got all the directors of allied health to agree on and yes they all have to collect them for their own department on a six monthly basis.” [R2 |
| **Research forums** | “…one of the things that we've set up with the research professor and the research committee, is a - twice a year we have a research and evidence-based practice symposium. “[M4]  “Also we have like an allied health grant round, which is actually - it's not a clinical grant round, it's research. So we ask each of our researchers who are published or about to publish to present their research project on a monthly basis. So that's been another success and that's held at lunchtime and we've had continued research workshops.” [M6] |
| **Resources and templates**  ***Mechanism- use group approach*** | “It's [Website] only been all there and up and running for a very short time. But in terms of, like I said, providing the podcasts of the education and training sessions, that series of lectures that we did, we can put them there as podcasts and then they're available to anybody to go to. A lot of it is very useful,…” [R4]  “Look necessity was the mother of invention in all of those situations it got to the point where I was being asked so frequently some of those questions that it was really just easier for me to develop the resources and distribute them out.” [R5]  “I guess a group approach to some of the documents. Yeah. It's probably more the group approach”. [R3] |

## 9. Research Position Development

| **Examples** | **Quotes** |
| --- | --- |
| **Developing networks and increasing knowledge** | “But I think the impact of the role on me has been quite incredible. …. it's amazing … how much you learn about the different disciplines and then develop those networks of how they work together or could work together. “ [R5]  “I mean I think it's been a great opportunity and obviously I've benefited from having this position, the investment by the Queensland Government into these roles.” R8 |
| **Inspiration and reward from seeing clinicians enthusiasm** | `“They’re just an awesome staff here and we just, we all benefit when we invest in them in that way.”[R8]  “…the enthusiasm that some of the staff approaches their research projects with are truly truly incredible. ... To watch that sort of thing you really that's what makes me try so hard in this role, seeing people like that.” [R5] |
| **Opportunity to make clinical changes** | “We are in such a lucky position, that the few barriers that we have are nothing, compared to what we are creating and what we're doing. Even in my lowest moments [laughs] of this job, you still think - yeah - it is totally, so worth it… it will only be giving good things for health.” [R7]  “I couldn't have made that happen in five years, being disconnected from Health and being just as a university academic. [R7] |
